# The value of unsupervised machine learning algorithms based on CT and MRI for predicting sarcopenia

**DOI:** 10.3389/fpubh.2025.1649400

**Published:** 2025-09-25

**Authors:** Huayan Zuo, Qiu Bi, Xiaolong Liu, Guoli Bi, Yijin Wang, Yunzhu Wu, Guang Yang, Chengxiu Zhang, Yang Song, Gang Wang, Qiyang Wang, Xiarong Gong

**Affiliations:** ^1^Department of MRI the First People’s Hospital of Yunnan Province, The Affiliated Hospital of Kunming University of Science and Technology, Kunming, China; ^2^Department of MRI, The First People’s Hospital of Yunnan Province, The Affiliated Hospital of Kunming University of Science and Technology, Kunming, China; ^3^Department of Medical Imaging, The People’s Hospital of Puer, The Affiliated Hospital of Kunming University of Science and Technology, Kunming, China; ^4^Institute for AI in Medicine, School of Artificial Intelligence, Nanjing University of Information Science and Technology, Nanjing, China; ^5^Shanghai Key Laboratory of Magnetic Resonance, East China Nornal University, Shanghai, China; ^6^MR Research Collaboration, Siemens Healthineers, Shanghai, China; ^7^Department of Radiology, the First People’s Hospital of Yunnan Province, The Affiliated Hospital of Kunming University of Science and Technology, Kunming, China; ^8^Department of Orthopedic Surgery, The Key Laboratory of Digital Orthopaedics of Yunnan Provincial, Yunnan Province Spinal Cord Disease Clinical Medical Center, The First People's Hospital of Yunnan Province, The Affiliated Hospital of Kunming University of Science and Technology, Kunming, China

**Keywords:** sarcopenia, computed tomography, magnetic resonance imaging, unsupervised machine learning, Gaussian mixture model

## Abstract

**Objectives:**

This study aims to investigate the efficacy of unsupervised machine learning algorithms, specifically the Gaussian Mixture Model (GMM), K-means clustering, and Otsu automatic threshold partitioning, in predicting sarcopenia based on computed tomography (CT) and magnetic resonance imaging (MRI) data.

**Methods:**

A retrospective analysis was conducted on a dataset comprising 191 patients diagnosed with sarcopenia and 327 control patients. Participants were randomly assigned to training and validation cohorts in a 6:4 ratio. The paravertebral muscles at the lumbar 3/4 intervertebral disc level were manually delineated as the region of interest (ROI) on non-enhanced CT and axial T2-weighted MRI images. Muscle and adipose tissues were automatically segmented from the ROI using GMM, K-means, and Otsu algorithms at the cohort level. Quantitative metrics such as mean, volume, and volume percentage were computed, and these parameters were compared between the sarcopenia and non-sarcopenia groups. Logistic regression analysis was employed to develop predictive models for sarcopenia, with model performance evaluated using the area under the curve (AUC). The stability of the models was assessed through five-fold cross-validation.

**Results:**

The study demonstrates that three unsupervised clustering algorithms utilizing CT data surpassed those employing MRI data. Notably, the CT-based Otsu model exhibited the highest predictive performance in both training and validation datasets, with AUC values of 0.986 and 0.958, respectively. This was followed by the CT-based GMM, which achieved AUC values of 0.990 and 0.903, and the K-means model, with AUC values of 0.727 and 0.772. Furthermore, the CT-based GMM model demonstrated superior stability upon five-fold cross-validation, yielding an average AUC of 0.990.

**Conclusion:**

The findings indicate that CT-based unsupervised machine learning models outperform their MRI-based counterparts, with the CT-based Otsu and GMM models showing exceptional efficacy in sarcopenia prediction, as evidenced by AUC values exceeding 0.95.

## Introduction

The European Working Group on Sarcopenia in Older People (EWGSOP) currently defines sarcopenia as “a syndrome characterized by progressive and generalized loss and change in skeletal muscle quality and strength” (1). It is an age-related disease characterized by a decline in muscle quality, strength, and function (2). From a clinical standpoint, the SARC-F questionnaire is a straightforward and well-established tool endorsed by the EWGSOP for identifying patients with compromised physical function and sarcopenia (3). However, diagnosing sarcopenia requires a variety of assessments, including muscle strength tests and more accurate imaging methods, often utilizing bioelectrical impedance analysis (BIA) or dual-energy X-ray absorptiometry (DXA) to evaluate muscle quality (4, 5). Dual-energy X-ray and bioelectrical impedance may underestimate the degree of muscle quality reduction, and factors such as hydration and water retention (such as heart, liver, or kidney failure) can affect the accuracy of these methods (6, 7). Additionally, many older population individuals experience conditions that lead to water retention, which can limit the applicability of certain assessment methods.

Accurate diagnosis of sarcopenia requires precise quantification of muscle including muscle quantity and quality. Computed tomography (CT) and magnetic resonance imaging (MRI) are considered the gold standards for non-invasive assessment of muscle (1, 8, 9). CT can directly analyze body composition and quantify muscle mass (10). Given that muscle density is associated with the extent of fat infiltration, CT can evaluate both muscle quantity and quality by accurately differentiating between fat and muscle tissues based on their specific attenuation characteristics, thereby providing detailed anatomical information (11). Over the past 25 years, the reliability of CT in assessing quantitative and qualitative changes in fat and muscle quality has been well demonstrated (12, 13). MRI also demonstrates high accuracy in assessing muscle and fat areas or volumes and in segmenting muscle on CT cross-sectional images (14). In addition to providing information on fatty infiltration similar to CT, MRI can also offer data on muscle edema, fiber infiltration, fiber contractility, and elasticity (15). However, these methods cannot provide precise measurements for evaluating changes over time, and no studies have compared CT and MRI for predicting sarcopenia. The majority of existing research predominantly employs supervised learning techniques, which necessitate substantial annotated datasets and exhibit high sensitivity to shifts in data distribution. In contrast to the prevalent focus on a single imaging modality (such as CT or MRI) in previous studies, this research uniquely integrates the strengths of both CT and MRI. CT is particularly effective for quantitative analyses of muscle density and fat infiltration, while MRI provides superior soft tissue contrast and detailed visualization of muscle fiber architecture.

To advance the exploration of sarcopenia, there is a pressing need for the development of precise quantitative assessment methodologies. Artificial intelligence (AI) holds potential to facilitate the integration of sarcopenia research, particularly concerning low muscle quantity and quality, into clinical practice (16). In recent years, the rapid advancement of AI technology has led to the increased application of unsupervised machine learning algorithms in the medical field, providing innovative strategies for sarcopenia prediction. Our preliminary investigations have demonstrated that unsupervised algorithms utilizing cohort-level clustering surpass those employing case-level clustering (17). In contrast to traditional radiomics, which necessitates large sample sizes for robust analysis, unsupervised machine learning algorithms exhibit reduced dependency on sample size. In the realm of medical image analysis, particularly in the assessment of muscle composition, there are significant challenges associated with acquiring large, high-quality labeled datasets due to financial and logistical constraints. Unsupervised learning algorithms address this limitation by identifying intrinsic data structures and patterns without the need for extensive annotations. This methodological approach is especially advantageous for studies on muscle composition where labeled data is scarce, as it reduces the dependency on annotations, lowers costs, and facilitates tissue segmentation and classification. These algorithms are capable of autonomously segmenting and classifying muscle tissues, as well as performing feature extraction and dimensionality reduction, thereby offering significant advantages. K-means clustering has been successfully applied to the automatic segmentation of muscle and fat (18). Although common unsupervised machine learning algorithms like the Gaussian mixture model (GMM) and Otsu thresholding are employed, there is limited literature on their application in muscle assessment.

This study aims to investigate the utility of unsupervised machine learning algorithms, such as Gaussian mixture modeling, K-means clustering, and Otsu thresholding, utilizing cohort-level CT and MRI data to predict sarcopenia.

## Materials and methods

### Study population

**Figure 1:**
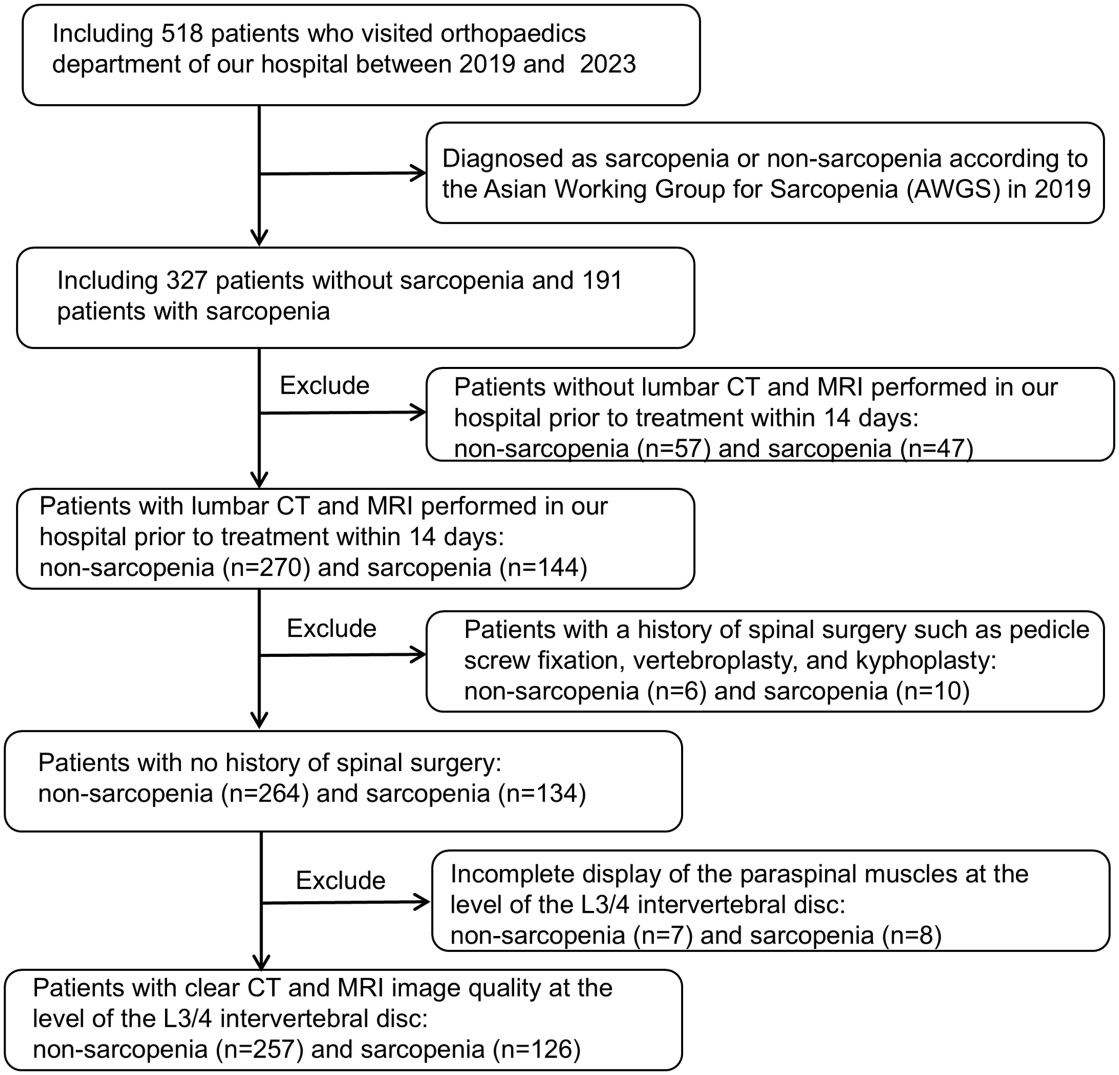
Flowchart of the patient collection process.

The study was conducted in accordance with the Declaration of Helsinki, and approved by the Institutional Review Board (or Ethics Committee) of the First People’s Hospital of Yunnan Province (reference number KHLL2023-KY209); date of approval 23 December 2024. The data are anonymous, and therequirement for informed consent was therefore waived. We consecutively reviewed 518 patients admitted to our hospital between August 2019 and October 2023, including 327 non-sarcopenia patients and 191 sarcopenia patients. And its validity was verified using prospectively collected data (65 non-sarcopenia patients and 36 sarcopenia patients) from our hospital between November 2023 and December 2024. Inclusion criteria: (1) patients diagnosed with sarcopenia or non-sarcopenia according to the Asian Working Group on Sarcopenia (AWGS) 2019 guidelines: Low Appendicular skeletal muscle mass (Dual-energy X-ray absorptiometry: male < 7.0 kg/m^2^, female: < 5.4 kg/m^2^ or Bioelectrical impedance analysis: male < 7.0 kg/m^2^, female < 5.7 kg/m^2^) and low muscle strength (Handgrip strength: male < 28 kg, female < 18 kg) or Low physical performance (6-metre walk: < 1.0 m/s or 5-time chair stand test: ≥ 12 s or Short Physical Performance Battery: ≤ 9); (2) age between 18 and 90 years old; (3) all patients had routine MRI and CT scans of the lumbar spine at our hospital within 14 days before treatment. Exclusion criteria: (1) patients who did not undergo lumbar non-enhanced CT or axial non-fat-suppressed T2-weighted imaging (T2WI) scanning; (2) patients who had undergone surgical treatment (pedicle screw fixation, vertebroplasty, and kyphoplasty); (3) poor-quality CT or MRI images, making evaluation impossible; (4) incomplete display of paraspinal muscles at the level of the L3/4 intervertebral disc. A total of 383 patients were finally enrolled, including 257 non-sarcopenia patients and 126 patients with sarcopenia. Clinical and laboratory data were retrospectively collected, encompassing variables such as age, gender, height, weight, body mass index (BMI), occupation, hypertension, diabetes, coronary heart disease, smoking history, preoperative red blood cell count, white blood cell count, serum albumin (ALB), hemoglobin, neutrophil count, neutrophil-to-lymphocyte ratio (NLR), and calcium concentration. Figure 1 shows the flow chart of patient inclusion and exclusion criteria.

### Imaging data acquisition

All retrospective patients MRI scans were performed using 1.5 T scanner (MAGNETOM Aera, Siemens Healthineers Ltd., Erlangen, Germany) and 1.5 T scanner (Ingenia, Philips, Netherlands) with 6- and 8-channel body coils, respectively. All patients underwent routine supine lumbar spine MRI scanning with the following sequences: lumbar spine sagittal T2/T1-weighted fast spin–echo sequences and lumbar intervertebral disc axial T2-weighted fast spin-echo sequences. The scan parameters are shown in Table 1. CT scans were performed using Somatom Definition, Aquilion One, Somatom Force, and Somatom Emotion 16 scanners with the following parameters: tube voltage = 100–120 kV, tube current = 20–500 mA, collimation degree = 40 mm, slice thickness = 3 mm, and pitch = 0.984:1. Some parameters were adjusted according to patients’ individual differences. All lumbar MRI scans of prospective patients were performed using a Siemens Aera 1.5 T MRI scanner (Germany), and all CT scans were conducted with a Siemens Somatom Force scanner, following standardized imaging protocols with identical scanning parameters as previously described.

**Table 1:** The details of MRI parameters of different sequences.

MRI scanning equipment and parameters		Repetition time (ms)	Echo time (ms)	Field of view (mm^2^)	Matrix	Slice thickness (mm)	Slice gap (mm)
Siemens Aera 1.5 T	Axial T2WI	3,100	94	210 × 210	384 × 288	4	0.4
	Sagittal T2WI	3,820	86	400 × 400	384 × 288	4	0.4
	Sagittal T1WI	644	9.5	400 × 400	384 × 288	4	0.4
Philips Ingenia 1.5 T	Axial T2WI	1,181	100	180 × 300	180 × 193	4	0.4
	Sagittal T2WI	180	100	180 × 300	200 × 248	4	0.4
	Sagittal T1WI	692	9	180 × 300	200 × 248	4	0.4

T2WI, T2-weighted image; T1WI, T1-weighted image.

### Image quantitative measurement

To preserve the original information from CT and MRI scans, images were stored in a lossless DICOM format. 3D Slicer software (version 5.6.2) and Python (version 3.11.4) were used to preprocess the images. The Resample Image (BRAINS) module resampled the DICOM images to a voxel size of 1 × 1 × 1 mm^3^, and N4 bias field correction reduced nonuniformity caused by different scanner magnetic fields. A radiologist with 2 years of experience in musculoskeletal imaging utilized MRI axial T2-weighted imaging (T2WI) sequences and CT axial images for anatomical segmentation at the L3-4 intervertebral disc level. The regions of interest (ROIs) for the studied muscles, including the bilateral paraspinal muscles and the psoas major muscle, were manually segmented. Prior to training any algorithm, a series of preprocessing steps were carried out on the ROIs within the training set. In this study, three unsupervised clustering methods—Otsu method, K-means, and GMM—were employed to distinguish muscle and fat tissues in the paraspinal muscles of the lumbar spine. The Otsu method maximized the inter-class variance to classify voxel intensity values into two categories and determine the optimal intensity threshold. K-means clustering used k-means++ initialization and iterative centroid optimization to assign voxels into two clusters representing muscle and fat. GMM was initialized based on the results of K-means and updated using the Expectation–Maximization (EM) algorithm. After clustering, we analyzed the intensity distribution to determine the thresholds for muscle and fat and generated histograms of intensity values to identify peak positions. The Otsu method was then used to optimize these thresholds. Finally, the obtained thresholds were applied to classify voxels in the test set ROIs. Subsequently, after the clustering process, the intensity distributions were analyzed to establish threshold values for muscle and fat. Histograms of intensity values were generated to pinpoint peak positions, and these thresholds were further refined using Otsu’s method.

Based on population-level clustering involves accumulating voxel matrices from all patients’ GMM, K-means, and Otsu clustering results into a global matrix. Using Python (3.9.12) for consistent clustering, we obtain voxel consistency patterns at the population level. Ultimately, habitat images for each patient based on population-level clustering are obtained. The open-source software FeAture Explorer (FAE, v0.5.16) is used to extract habitat parameters, including the volume, percentage, and voxel mean across for each habitat.

To evaluate the reliability of intra-observer and inter-observer delineation of the ROI, a second independent delineation was performed 3 months later on a randomly selected cohort of 50 patients. This task was executed by two radiologists: one who had previously delineated the ROI and another with 15 years of expertise in musculoskeletal diagnostics.

### Model building

**Figure 2:**
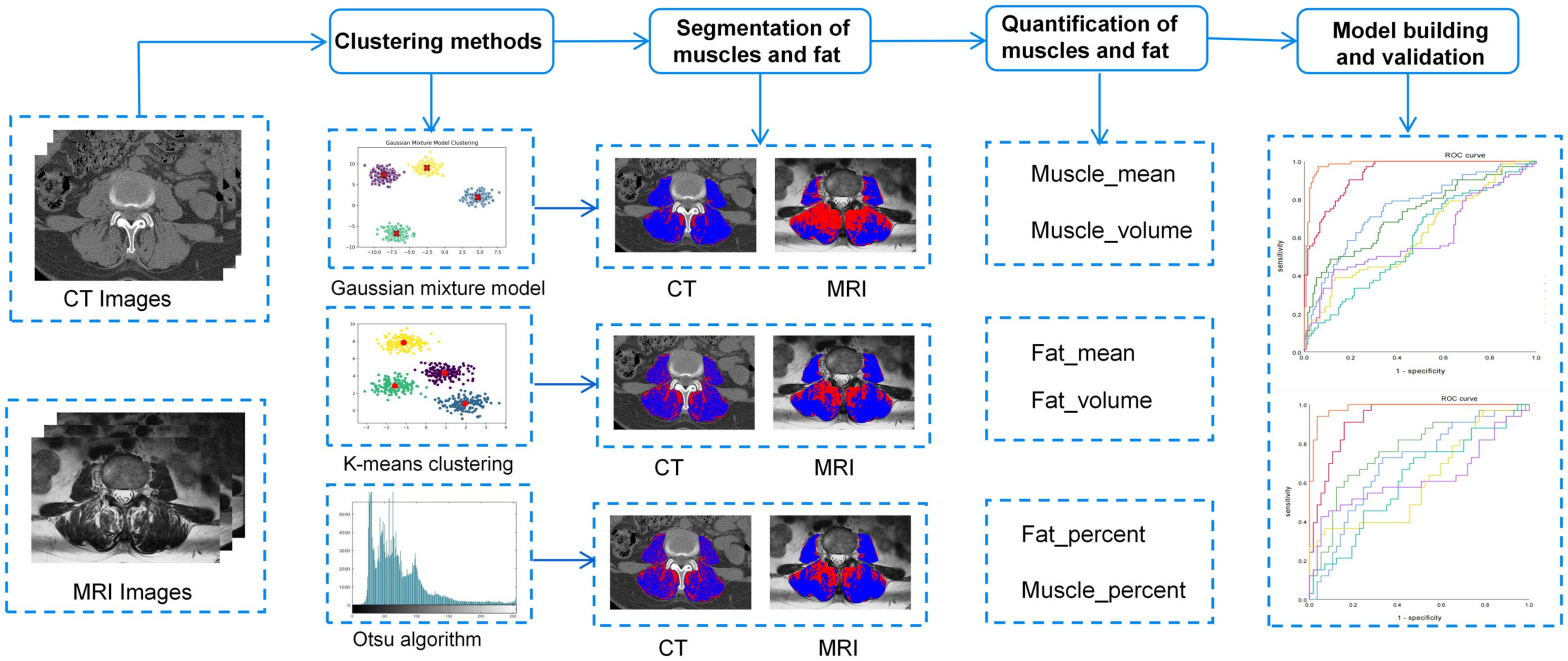
The overall workflow of this study.

Initially, univariate analysis was employed to compare the characteristics of patients with and without sarcopenia within the training cohort, aiming to identify parameters with statistically significant differences. Subsequently, individual predictors for sarcopenia were selected based on univariate logistic regression analysis. A predictive model was then developed using multivariate logistic regression, incorporating efficient predictive parameters. Various models were constructed to predict sarcopenia utilizing three unsupervised machine learning algorithms: CT-based GMM, K-means, and Otsu models, as well as MRI-based GMM, K-means, and Otsu models. Additionally, a separate clinical model was developed. A flowchart of the research process is shown in Figure 2.

### Statistical analysis

The study population was randomly divided into training and retrospective validation sets in a 6:4 ratio using Python (version 3.12.4). Data were analyzed using SPSS statistical software (version 26.0 for Windows, IBM) and GraphPad Prism 9.5. A *p*-value less than 0.05 was considered statistically significant. A two-way random effects model, along with the Intraclass Correlation Coefficient (ICC), was utilized to assess the consistency of intra-observer and inter-observer measurement outcomes. The normality of the data was evaluated using the Kolmogorov–Smirnov test, with continuous variables reported as mean ± standard deviation. To compare differences in quantitative parameters such as age, height, weight, and BMI between the non-sarcopenia and sarcopenia groups in both the training and validation datasets, independent sample t-tests (for normally distributed data with homogeneity of variance) or Mann–Whitney U tests, along with chi-square and Fisher’s exact tests (for categorical variables), were employed. Statistically significant indicators were identified. In the training dataset, univariate logistic regression analysis and multivariate logistic regression with backward stepwise selection were conducted to identify independent predictors and to develop a predictive model for sarcopenia. The predictive performance of the models was evaluated and compared using the area under the receiver operating characteristic (ROC) curve (AUC), from which the corresponding AUC, optimal cutoff value, sensitivity, specificity, and accuracy were derived. The stability of each model was assessed with a five-fold cross-validation ROC curve. The calculation formulas for part of the data in this study are as follows: Sensitivity: Alias: True Positive Rate (TPR). The sensitivity formula is: Sensitivity = TP/(TP + FN). Specificity: Alias: True Negative Rate (TNR). The specificity formula is: Specificity = TN/(TN + FP). The accuracy formula is: Accuracy = (TP + TN)/(TP + TN + FP + FN). TP (true positive); FP (false positive); FN (false negative); TN (true negative). The workflow of this study is illustrated in Supplementary Figure S1.

## Results

### Clinical parameters

The clinical characteristics of the subjects in the training and retrospective validation sets are summarized in Table 2. A total of 383 patients were divided into a training set (228 patients) and a retrospective validation set (155 patients). In the training set, 149 (65.35%) were non-sarcopenia patients and 79 (34.65%) were sarcopenia patients. In the retrospective validation set, there were 108 non-sarcopenia patients (69.68%) and 47 sarcopenia patients (30.32%). The mean age of the sarcopenia group in the training set (71.65 ± 7.01 years) was greater than that of the non-sarcopenia group (68.13 ± 6.88 years). The statistical analysis revealed no significant differences between the non-sarcopenia and sarcopenia groups concerning variables such as gender, occupation, hypertension, diabetes mellitus, coronary heart disease, pulmonary disease, smoking history, preoperative white blood cell count, neutrophil count, NLR, and calcium ion concentration, with all *p*-values exceeding 0.05. However, significant differences were observed in age, BMI, preoperative RBC, hemoglobin, and serum albumin (all *p* < 0.05). According to univariate and multivariate logistic regression analysis, age, BMI, and preoperative serum albumin were effective predictive parameters, as shown in Table 3.

**Table 2:** Demographic and clinical characteristics of the training and validation groups.

Clinical features	Training group (*n* = 228)			Retrospective validation group (*n* = 155)			Prospective validation group (*n* = 101)		
	Non-sarcopenia (*n* = 149)	Sarcopenia (*n* = 79)	*p*	Non-sarcopenia (*n* = 108)	Sarcopenia (*n* = 47)	*p*	Non-sarcopenia (*n* = 65)	Sarcopenia (*n* = 36)	*p*
Age (years)	68.13 ± 6.88	71.65 ± 7.01	<0.001*	67.20 ± 5.81	70.77 ± 5.90	0.001*	67.43 ± 5.91	71.28 ± 7.22	0.015*
Gender (Female/Male)	88/61	45/34	0.760	60/48	35/12	0.026*	34/31	24/12	0.140
Career									
Worker	23	7	0.681	35	7	0.077	13	12	0.011*
Retired	22	13		14	12		11	1	
Farmer	27	13		32	12		27	14	
Freelance	3	2		5	2		12	3	
Others	74	44		22	14		2	6	
Hypertension (yes/no)	74/75	37/42	0.622	42/66	14/33	0.278	23/42	20/16	0.050
Diabetes (yes/no)	21/128	11/68	0.972	18/90	7/40	0.783	6/59	2/34	0.787
Smoking (yes/no)	31/118	20/59	0.437	18/90	5/42	0.332	18/47	6/30	0.212
Pulmonary Disease (yes/no)	10/139	10/69	0.131	7/101	2/45	0.864	1/64	1/35	1.000
Cardiac Disease (yes/no)	15/134	14/65	0.099	11/97	4/43	0.977	1/64	2/34	0.598
BMI (kg/m^2^)	24.99 ± 3.40	22.83 ± 3.18	<0.001*	22.11 ± 6.62	23.41 ± 3.03	0.963	23.99 ± 5.36	22.78 ± 3.53	0.014*
WBC (10^9^/L)	6.23 ± 1.83	6.27 ± 2.06	0.992	6.13 ± 2.06	5.91 ± 1.89	0.664	6.00±1.72	7.00 ± 2.75	0.128
NEUT (10^9^/L)	3.65 ± 1.57	3.83 ± 1.57	0.451	3.63 ± 1.72	3.58 ± 1.52	0.958	3.58 ± 1.38	4.28 ± 2.43	0.236
NLR	1.84 ± 0.55	1.80 ± 0.85	0.072	2.02 ± 1.22	2.24 ± 1.02	0.231	1.76 ± 0.30	1.76 ± 0.33	0.999
RBC (10^12^/L)	4.50 ± 0.54	4.36 ± 0.48	0.009*	4.49 ± 0.49	4.34 ± 0.54	0.098	4.49 ± 0.50	4.26 ± 0.45	0.025*
Calcium (mmol/L)	2.15 ± 0.12	2.13 ± 0.10	0.545	4.49 ± 0.49	4.34 ± 0.54	0.167	2.15 ± 0.09	2.13 ± 0.11	0.915
Hemoglobin (g/L)	136.76 ± 18.03	130.77 ± 16.31	0.009*	135.79 ± 15.14	128.85 ± 16.31	0.011*	138.89 ± 16.14	131.00 ± 12.66	0.013*
Albumin (g/L)	37.89 ± 3.09	35.90 ± 3.32	<0.001*	39.25 ± 3.45	37.48 ± 2.94	0.003*	38.46 ± 3.57	37.66 ± 3.78	0.476

Data are mean  ±  standard deviation; BMI, body mass index; WBC, white blood cell; NEUT, neutrophil; NLR, Neutrophil-to-Lymphocyte; RBC, red blood cell. *p < 0.05.

**Table 3:** Logistic regression analysis of clinical characteristics between the non-sarcopenia patients and the sarcopenia patients in the training group.

Clinical features	Univariable LR		Multivariable LR	
	OR (95% CI)	*p*	OR (95% CI)	*p*
Age (years)	1.07 (1.03–1.12)	0.001*	1.07 (1.02–1.12)	0.004*
BMI (kg/m^2^)	0.81 (0.73–0.89)	<0.001*	0.82 (0.73–0.91)	<0.001*
RBC (10^12^/L)	0.58 (0.33–1.00)	0.051	1.31 (0.47–3.63)	0.605
HGB (g/L)	0.98 (0.96–1.00)	0.017*	0.99 (0.96–1.02)	0.678
ALB (g/L)	0.82 (0.75–0.90)	<0.001*	0.86(0.78–0.96)	0.007*

LR, Logistic regression; OR, odds ratio; CI, confidence interval; NA, not applicable. *p < 0.05.

### Intra- and inter-observer consistency of quantitative parameters

The quantitative parameters derived from a single radiologist delineating the ROI twice demonstrated strong intra-observer reliability, while those obtained from two radiologists independently delineating the ROI exhibited robust inter-observer reliability. Specifically, for ROI delineation in CT images, the intra-observer and inter-observer ICCs were 0.976 and 0.928, respectively. For ROI delineation in MRI images, the intra-observer and inter-observer ICCs were 0.994 and 0.945, respectively. Detailed results are presented in Supplementary Figure S2.

### Image analysis

**Figure 3:**
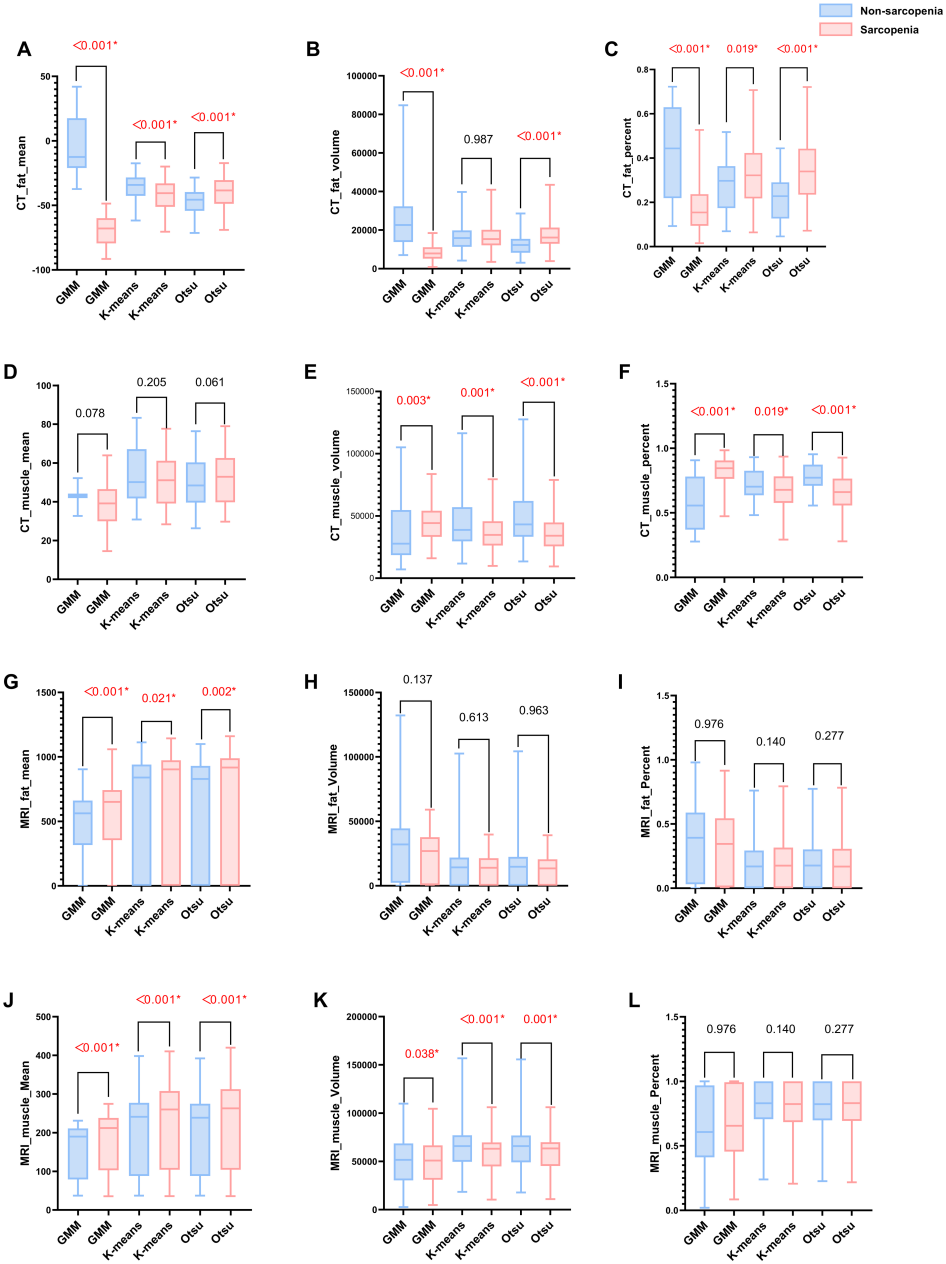
**(A–L)** Bar graphs of MRI and CT characteristics derived from different models in the non-sarcopenia patients and the sarcopenia patients in the training group. A red asterisk indicate statistical significance difference.

Within the training set, the differences in mean, volume, and percentage of fat, as well as in volume and percentage of muscle, between the non-sarcopenia and sarcopenia groups were statistically significant (*p* < 0.05) in the CT-based GMM. Similar significant differences were observed in the CT-based Otsu models. The differences in mean, and percentage of fat and in volume, and percentage of muscle between the non-sarcopenia and sarcopenia groups were statistically significant (*p* < 0.05) in the CT-based K-means model. In the MRI-based GMM model, differences in mean of fat and differences in mean, volume of muscle were statistically significant (*p* < 0.05). Similar significant differences were observed in the MRI-based K-means and Otsu models. The analysis results are shown in Figure 3 and Supplementary Table 1. Independent predictors in the CT-based GMM model was fat mean. In the CT-based K-means model, the predictors were fat mean and percentage, and muscle volume and percentage. In the CT-based Otsu model, the predictors were fat mean and percentage, and muscle volume and percentage. In the MRI-based GMM model, the predictor was muscle mean; in the MRI-based K-means model, it was muscle volume; and in the MRI-based Otsu model, it was muscle volume, as shown in Table 4.

**Table 4:** Logistic regression analysis of different imaging models between non-sarcopenia patients and the sarcopenia patients in the training group.

Different models		Univariable LR		Multivariable LR	
		OR (95% CI)	*p*	OR (95% CI)	*p*
GMM	CT_fat_Mean	0.81 (0.75–0.89)	<0.001*	0.81 (0.74–0.89)	<0.001*
	CT_fat_Volume	1.00 (1.00–1.00)	<0.001*	1.00 (1.00–1.00)	0.625
	CT_muscle_Volume	1.00 (1.00–1.00)	0.136	NA	NA
	CT_fat_Percent	0.00 (0.00–0.00)	<0.001*	3.58 (0.00–116816.27)	0.810
	CT_muscle_Percent	7033.60 (423.61–116785.08)	<0.001*	3.58 (0.00–116816.27)	0.810
Kmeans	CT_fat_Mean	0.94 (0.91–0.97)	<0.001*	0.89 (0.84–0.94)	<0.001*
	CT_muscle_Volume	1.00 (1.00–1.00)	0.001*	1.00 (1.00–1.00)	0.003*
	CT_fat_Percent	17.70(1.59–197.11)	0.019*	0.00 (0.00–0.00)	<0.001*
	CT_muscle_Percent	0.06 (0.01–0.63)	0.019*	40846.54 (113.67–14677590.73)	<0.001*
Otsu	CT_fat_Mean	1.08 (1.05–1.11)	<0.001*	1.74 (1.43–2.10)	<0.001*
	CT_fat_Volume	1.00 (1.00–1.00)	<0.001*	1.00 (1.00–1.00)	0.125
	CT_muscle_Volume	1.00 (1.00–1.00)	<0.001*	1.00 (1.00–1.00)	0.040*
	CT_fat_Percent	15150.84 (755.29–303920.42)	<0.001*	6.58 (5.64–7.68)	<0.001*
	CT_muscle_Percent	0.00 (0.00–0.00)	<0.001*	0.00 (0.00–0.00)	<0.001*
GMM	MRI_fat_Mean	1.00 (1.00–1.00)	0.005*	1.00 (1.00–1.00)	0.745
	MRI_muscle_Mean	1.00 (1.00–1.01)	0.002*	1.01 (1.00–1.02)	0.015*
	MRI_muscle_Volume	1.00 (1.00–1.00)	0.038*	1.00 (1.00–1.00)	0.478
Kmeans	MRI_fat_Mean	1.00 (1.00–1.00)	0.430	NA	NA
	MRI_muscle_Mean	1.00 (1.00–1.01)	0.021*	1.00 (1.00–1.00)	0.922
	MRI_muscle_Volume	1.00 (1.00–1.00)	0.001*	1.00 (1.00–1.00)	0.010*
Otsu	MRI_fat_Mean	1.00 (1.00–1.00)	0.322	NA	NA
	MRI_muscle_Mean	1.00 (1.00–1.01)	0.010*	1.00 (1.00–1.00)	0.458
	MRI_muscle_Volume	1.00 (1.00–1.00)	0.002*	1.00 (1.00–1.00)	0.044*

GMM, Gaussian mixture model. *p < 0.05.

### The performance of different models and cross-validation on the training data set

**Figure 4:**
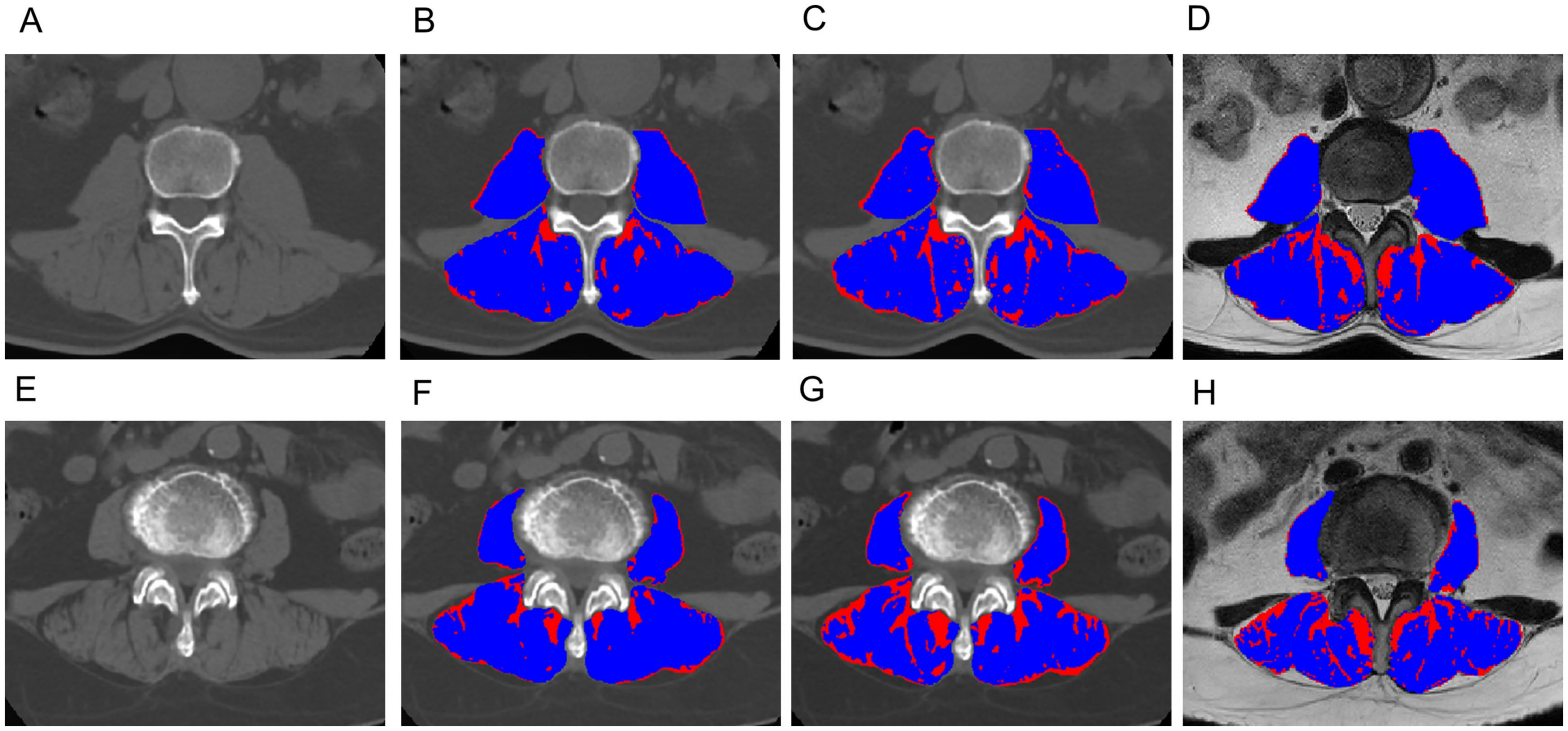
Exemplary slices showing the differentiation between muscle (blue) and fat (red) obtained with the CT-based Gaussian Mixture Model **(B,F)**, CT-based Otsu thresholding method **(C,G)** and MRI-based Otsu thresholding method **(D,H)**, **(A–D)**: A 62-year-old male with sarcopenia; **(E–H)**: an 66-year-old female without sarcopenia. The AUC of the CT-based Gaussian Mixture Model and the Otsu model for predicting sarcopenia was 0.98 and 0.93, respectively. The AUC of the Gaussian mixture model and the Otsu model for predicting sarcopenia was 0.990 and 0.986, respectively.

**Figure 5:**
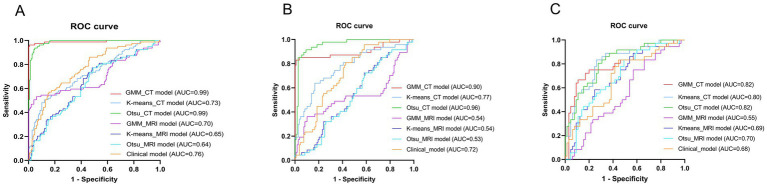
Receiver operator characteristic (ROC) curves of different models in the training group **(A)**, the retrospective validation group **(B)** and the prospective validation group **(C)**.

Table 5 presents the comparison of the diagnostic efficiency of different models. In the training set, the CT-based GMM model achieved the highest AUC values (AUC = 0.990), followed by the CT-based Otsu model (AUC = 0.986), the clinical model (AUC = 0.764), the CT-based K-means model (AUC = 0.727), the MRI-based GMM model (AUC = 0.697), the MRI-based K-means model (AUC = 0.649), and the MRI-based Otsu model (AUC = 0.638). In the retrospective validation group, the diagnostic efficiency from highest to lowest was: CT-based Otsu model (AUC = 0.958), CT-based GMM model (AUC = 0.903), the CT-based K-means model (AUC = 0.772) clinical model (AUC = 0.719), MRI-based K-means model (AUC = 0.537), MRI-based GMM model (AUC = 0.537), and MRI-based Otsu model (AUC = 0.528). In the prospective validation cohort, the CT-based Otsu model demonstrated the highest diagnostic efficiency (AUC = 0.819). Figure 4 shows a schematic of the segmentation for the better-performing models, and Figure 5 presents the ROC curves for model comparison. Using five-fold cross-validation on the training set, the CT-based GMM model demonstrated the best stability (average AUC = 0.99), followed by the CT-based Otsu model (average AUC = 0.94). Detailed ROC curves and average AUC values for the five-fold cross-validation are shown in Supplementary Figures S3, S4.

**Table 5:** Comparison of AUCs for different models in the training group and the validation group.

Groups	Models	AUC (95% CI)	Sensitivity	Specificity	Youden index	Cut-off values
Training group	Clinical model	0.764 (0.699–0.828)	57.0%	84.6%	0.416	0.429
	GMM_CT	0.990 (0.975–1.000)	96.2%	99.3%	0.955	0.236
	Kmeans_CT	0.727 (0.656–0.797)	50.6%	87.9%	0.385	0.484
	Otsu_CT	0.986 (0.975–0.997)	93.7%	95.3%	0.890	0.438
	GMM_MRI	0.697 (0.616–0.779)	53.2%	94.0%	0.472	0.432
	Kmeans_MRI	0.649 (0.574–0.724)	77.2%	51.7%	0.289	0.309
	Otsu_MRI	0.638 (0.562–0.713)	77.2%	51.0%	0.282	0.313
Retrospective validation group	Clinical model	0.719 (0.639–0.800)	80.9%	57.4%	0.383	0.265
	GMM_CT	0.903 (0.833–0.972)	83.0%	99.1%	0.821	0.368
	Kmeans_CT	0.772 (0.688–0.856)	63.8%	83.3%	0.471	0.400
	Otsu_CT	0.958 (0.927–0.990)	85.1%	97.2%	0.823	0.522
	GMM_MRI	0.537 (0.424–0.650)	89.8%	36.2%	0.260	0.274
	Kmeans_MRI	0.537 (0.442–0.633)	72.3%	41.7%	0.140	0.290
	Otsu_MRI	0.528 (0.432–0.623)	72.3%	40.7%	0.130	0.292
Prospective validation group	Clinical model	0.675 (0.575–0.765)	83.33%	55.38%	0.387	0.315
	GMM_CT	0.817 (0.727–0.887)	83.33%	73.85%	0.572	0.370
	Kmeans_CT	0.801 (0.710–0.874)	66.67%	89.23%	0.559	0.365
	Otsu_CT	0.819 (0.730–0.889)	80.56%	72.31%	0.529	0.391
	GMM_MRI	0.550 (0.448–0.650)	75.00%	43.08%	0.181	0.349
	Kmeans_MRI	0.693 (0.594–0.781)	58.33%	75.38%	0.337	0.402
	Otsu_MRI	0.695 (0.596–0.783)	83.33%	50.77%	0.341	0.323

AUC, area under the curve.

## Discussion

In this study, we introduce an innovative unsupervised learning algorithm designed for the quantitative assessment of paraspinal muscle components utilizing CT and MR imaging modalities. Our findings indicate that the Otsu and GMM algorithms, when applied to CT scans, exhibit superior efficacy in predicting sarcopenia. Through logistic regression analysis, we determined that age, BMI, and serum albumin levels are significantly associated with sarcopenia. It is well-documented that the prevalence of sarcopenia escalates with advancing age, and its implications for public health are intensifying due to the aging population (19). BMI is a critical factor in sarcopenia (20). BMI emerges as a pivotal factor in the context of sarcopenia, as weight gain and obesity can expedite the onset and progression of this condition, either directly or indirectly. In individuals with obesity, the accumulation of pro-inflammatory macrophages and other immune cells within adipose tissue, coupled with the dysregulated secretion of various adipokines and cytokines, fosters a chronic pro-inflammatory state (21, 22). Furthermore, the excessive secretion from adipose tissue and the compromised lipid storage capacity observed in obese individuals contribute to ectopic lipid deposition within skeletal muscle. Intramuscular lipids contribute to mitochondrial dysfunction and enhance the secretion of specific pro-inflammatory myokines, thereby inducing muscle dysfunction (23). It is hypothesized that individuals with higher adiposity may have increased protein intake, which serves as a protective factor against sarcopenia (24). Consequently, maintaining a healthy weight is essential for preserving muscle mass and strength in older adults. Serum albumin concentration is a valuable indicator of an individual’s nutritional status, with lower levels signifying reduced protein reserves and the initiation of catabolic processes that lead to muscle degradation (25–27). Elevated free cortisol levels have been observed in patients with hypoalbuminemia, further promoting muscle breakdown, particularly in inactive individuals. Albumin is capable of activating the phosphatidylinositol 3-kinase pathway, which mediates muscle. This study used measurement of muscle mass at the level of the L3 because the muscle mass at the L3 vertebral level is recognized as a pivotal indicator for evaluating total body muscle mass. The cross-sectional area (CSA) at this level demonstrates a robust correlation with overall body muscle mass and serves as an effective reflection of the systemic musculature’s health status. Numerous clinical guidelines and studies have identified the skeletal muscle cross-sectional area (SMCSA) at the L3 level as a critical metric for assessing sarcopenia (28).

In our study, the predictive performance of CT-based models was superior to that of MRI-based models. CT has emerged as the most widely utilized cross-sectional imaging modality globally. Fron et al. (10) demonstrated a high level of concordance in imaging-derived biomarkers of muscle quantity and mass between CT and MRI in healthy subjects, indicating that both modalities can be used interchangeably for skeletal muscle assessment. The muscle area calculated from CT scans exhibits a strong correlation with total body muscle mass and is regarded as the gold standard for body composition analysis and the diagnosis of abnormal body composition phenotypes, particularly in malnourished patients (29–31). Tandon et al. observed that skeletal muscle area measurements obtained directly from CT and MRI tend to be systematically larger in CT than in MRI (32, 33). CT is capable of accurately quantifying muscle quality and identifying intramuscular fat, making it suitable for assessing muscle steatosis (34). Quantitative tissue measurements from CT are highly reproducible and show a strong correlation with clinical outcomes (35). CT offers advantages in the staging and follow-up of cancer and other diseases, rendering it ideal for the opportunistic evaluation of sarcopenia without the need for additional tests. Consequently, CT is extensively utilized in research across several retrospective and prospective analyses (36).

MRI holds promise for the assessment of sarcopenia; however, its application is constrained by high costs, extended scanning and post-processing durations, operational complexity, and the absence of standardized protocols. The quality of MRI imaging is highly contingent upon the configuration of scanning parameters. Inappropriate selection of parameters, such as echo time (TE), repetition time (TR), may lead to suboptimal image contrast or increased noise, thus compromising the clear visualization of soft tissues. In addition, this study segmented muscle and fat in the paraspinal muscles on T2WI and CT. The soft tissue contrast of MRI is not as clear as that of CT, so its predictive performance for sarcopenia is lower than that of CT. Consequently, its clinical use is largely confined to scenarios involving disease treatment or follow-up (37). The Otsu automatic thresholding algorithm, which segments images into foreground and background by optimizing inter-class variance based on the gray-level histogram, has been employed by Rui et al. to segment muscle tissue and quantify the fatty content of the gluteus maximus. This method yielded Dice Similarity Coefficients (DSCs) of 0.930 and 0.873, with fat ratio measurements aligning closely with those of radiologists, thereby demonstrating robust segmentation performance and potential for further muscle assessment (38, 39). In our study, the Otsu thresholding technique exhibited strong predictive capabilities, effectively delineating muscle tissue contours and features pertinent to sarcopenia prediction.

Additionally, the GMM, a renowned segmentation method grounded in mixture models, provides flexibility and efficacy for multivariate data analysis, and is frequently employed by researchers to address critical challenges in image segmentation. Ryan et al. (40) employed the GMM for the segmentation of abdominal organs, achieving DSC values ranging from 0.7 to 0.9, thereby establishing a benchmark for large-scale automatic abdominal segmentation. In our research, the GMM also exhibited superior predictive efficacy, suggesting that among the models evaluated, the GMM holds greater utility for assessing sarcopenia. This phenomenon may be attributed to the GMM’s capability to partition data into distinct Gaussian distributions by identifying these distributions within the dataset, thus enabling the model to adapt to real-world data with diverse cluster structures (41).

Conversely, K-means clustering, a fundamental clustering technique known for its straightforward structure, rapid segmentation, and versatility, was found by Thomas et al. (13) to effectively quantify total muscle volume compartments in sarcopenia, successfully distinguishing between contractile and non-contractile tissue components. However, in our study, the performance of K-means was suboptimal, potentially due to challenges in determining initial cluster centers and the slow convergence speed when processing large-scale images (42).

This study has some limitations. Firstly, this study is a single-center retrospective analysis with a limited sample size and lacks external validation. Although we have employed five-fold cross-validation to verify our findings, future research should involve multi-center studies with larger sample sizes. Secondly, despite standardizing image processing, variations in MRI scanning parameters, such as slice thickness, exist among different patients. The impact of these variations on our analysis remains uncertain, and we intend to conduct prospective validation in the future. Thirdly, the ROIs in this study were manually delineated by a single operator. While manual sketching is considered the gold standard in research, it is time-consuming and susceptible to the operator’s experience. Future studies should explore more accurate automatic or semi-automatic methods for lesion delineation.

## Conclusion

Unsupervised clustering algorithms based on CT and MRI have demonstrated effectiveness in predicting sarcopenia, with the CT-based Otsu and GMM exhibiting superior accuracy, achieving an AUC greater than 0.95. This model can be widely used in primary healthcare settings or developing regions with aging populations and can assist healthcare providers in treating patients with early-stage sarcopenia. Our research underscores the importance of imaging modalities (CT and MRI) in diagnosing sarcopenia. Integrating these evaluations with clinical symptoms and physical exams can improve diagnostic accuracy and patient care. For prevention, lifestyle interventions are crucial. Healthcare professionals should promote regular exercise, balanced diets, and healthy habits through comprehensive health education. Implementing these insights in healthcare policies and practices can enhance outcomes for sarcopenia patients, reduce healthcare burdens, and improve quality of life for the aging population.

## Data Availability

The raw data supporting the conclusions of this article will be made available by the authors, without undue reservation.
