# Supplementary material for: The value of unsupervised machine learning algorithms based on CT and MRI for predicting sarcopenia
Source: Front Public Health. 2025 Sep 25;13:1649400. doi: 10.3389/fpubh.2025.1649400 (PMC12507879; doi:10.3389/fpubh.2025.1649400)
Supplement: Supplementary file 1 [file Supplementary_file_1.doc]

**Supplementary Table 1.** Comparison of CT and MRI characteristics derived from different models between non-sarcopenia patients and sarcopenia patients in the training group.

| Different models | | Non-sarcopenia (n= 149) | Sarcopenia (n=79) | *P* |
| --- | --- | --- | --- | --- |
| GMM | CT_fat_Mean  CT_fat_Volume  CT_muscle_Mean  CT_muscle_Volume  CT_fat_Percent  CT_muscle_Percent | -2.66±22.51  23838.59±11691.02  43.21±3.31  39236.93±24710.70  0.42±0.21  0.58±0.21 | -67.65±15.46  9232.06±5663.38  40.38±10.42  43775.32±14499.66  0.18±0.10  0.82±0.10 | <0.001*  <0.001*  0.078  0.003*  <0.001*  <0.001* |
|  |
| K-means | CT_fat_Mean  CT_fat_Volume  CT_muscle_Mean  CT_muscle_Volume  CT_fat_Percent  CT_muscle_Percent | -36.30±9.58  15950.60±6306.24  54.00±14.04  47124.91±22331.81  0.28±0.12  0.72±0.12 | -42.70±10.60  15936.99±6041.91  52.06±13.42  37070.39±14543.39  0.31±0.11  0.69±0.11 | <0.001*  0.987  0.205  0.001*  0.019*  0.019* |
| Otsu | CT_fat_Mean  CT_fat_Volume  CT_muscle_Mean  CT_muscle_Volume  CT_fat_Percent  CT_muscle_Percent | -47.50±9.18  12286.00±5236.44  49.51±12.34  50789.51±22741.89  0.21±0.10  0.79±0.10 | -40.43±10.72  16721.18±6212.17  53.19±12.34  36286.20±14494.17  0.33±0.12  0.67±0.12 | <0.001*  <0.001*  0.061  <0.001*  <0.001*  <0.001* |
| GMM | MRI_fat_Mean  MRI_fat_Volume  MRI_muscle_Mean  MRI_muscle_Volume  MRI_fat_Percent  MRI_muscle_Percent | 517.05±182.88  26950.39±24794.69  149.32±66.67  54280.85±26192.95  0.33±0.29  0.67±0.29 | 594.73±209.29  24563.77±20730.46  180.32±75.45  46784.28±24620.14  0.36±0.30  0.64±0.30 | <0.001*  0.137  <0.001*  0.038*  0.976  0.976 |
| K-means | MRI_fat_Mean  MRI_fat_Volume  MRI_muscle_Mean  MRI_muscle_Volume  MRI_fat_Percent  MRI_muscle_Percent | 558.74±444.27  13647.79±14999.60  190.91±99.14  67583.41±21396.11  0.17±0.18  0.83±0.18 | 608.97±482.56  14072.71±12686.42  224.51±110.53  57275.30±19511.56  0.21±0.19  0.79±0.19 | 0.021*  0.613  0.001*  <0.001*  0.140  0.140 |
| Otsu | MRI_fat_Mean  MRI_fat_Volume  MRI_muscle_Mean  MRI_muscle_Volume  MRI_fat_Percent  MRI_muscle_Percent | 556.33±437.41  13986.56±15333.99  189.47±97.81  67244.66±21558.31  0.17±0.18  0.83±0.18 | 619.21±490.59  13548.96±12266.78  227.43±112.96  57799.10±19254.34  0.20±0.18  0.80±0.18 | 0.002*  0.963  <0.001*  0.001*  0.277  0.277 |

*: *P*<0.05


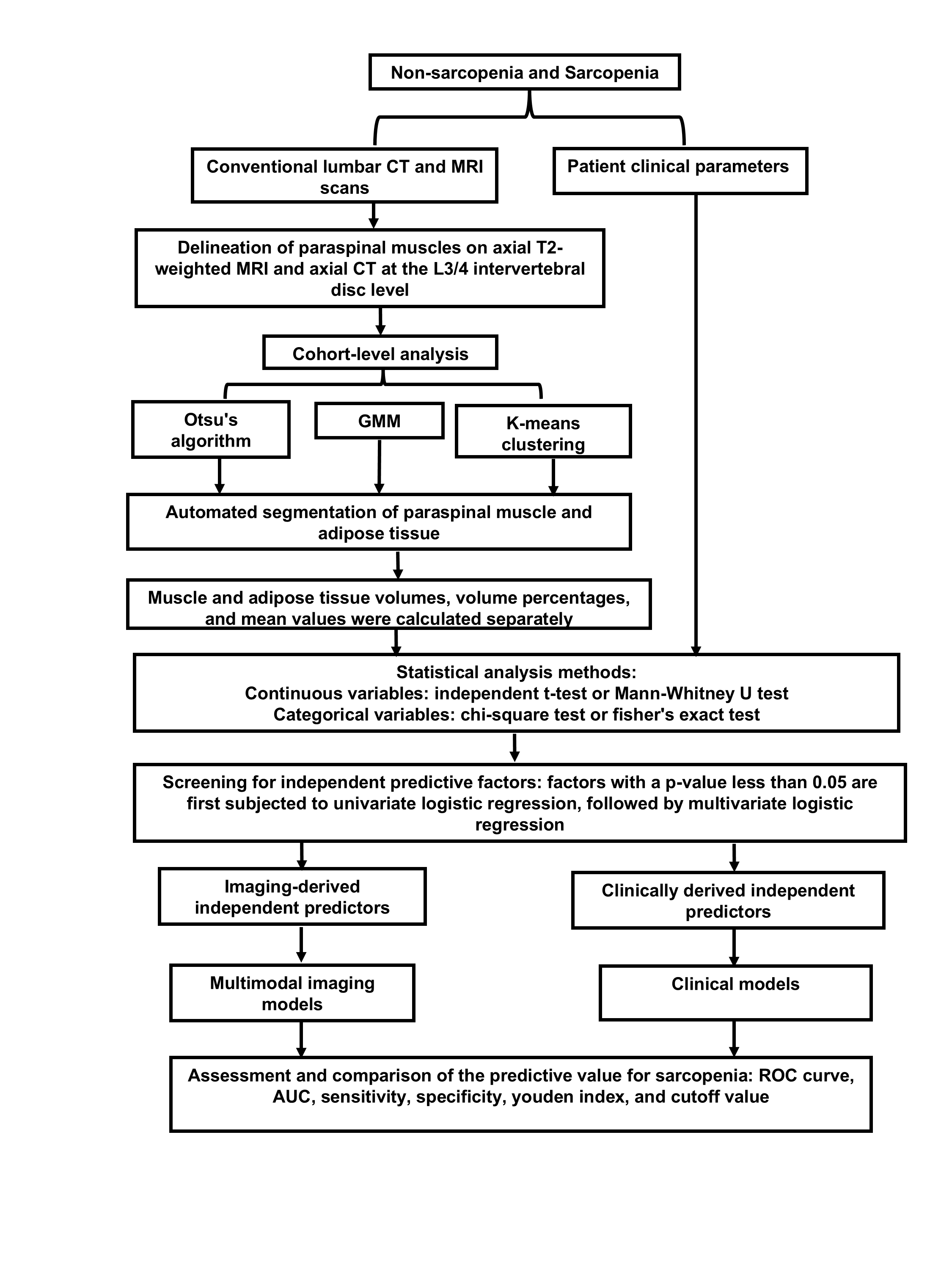


**Supplementary Figure S1.** The workflow diagram of this study.


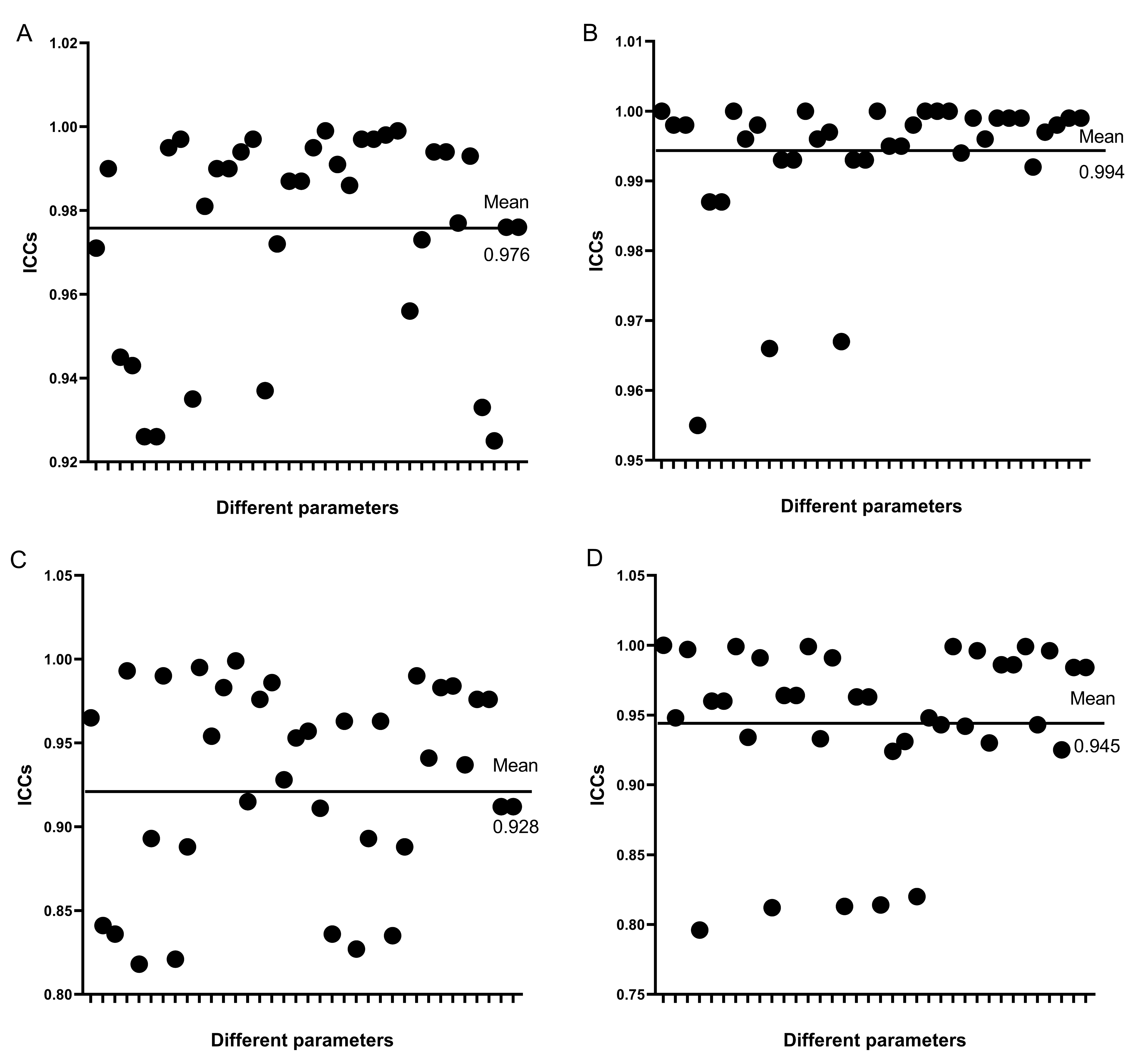


**Supplementary Figure S2.** Scatter grams show intra-observer and inter-observer consistency test results.

1. Intra-observer ICC values were calculated for quantitative parameters based on CT imaging.
2. Intra-observer ICC values were calculated for quantitative parameters based on MRI imaging.
3. Iner-observer ICC values were calculated for quantitative parameters based on CT imaging.
4. Inter-observer ICC values were calculated for quantitative parameters based on MRI imaging.


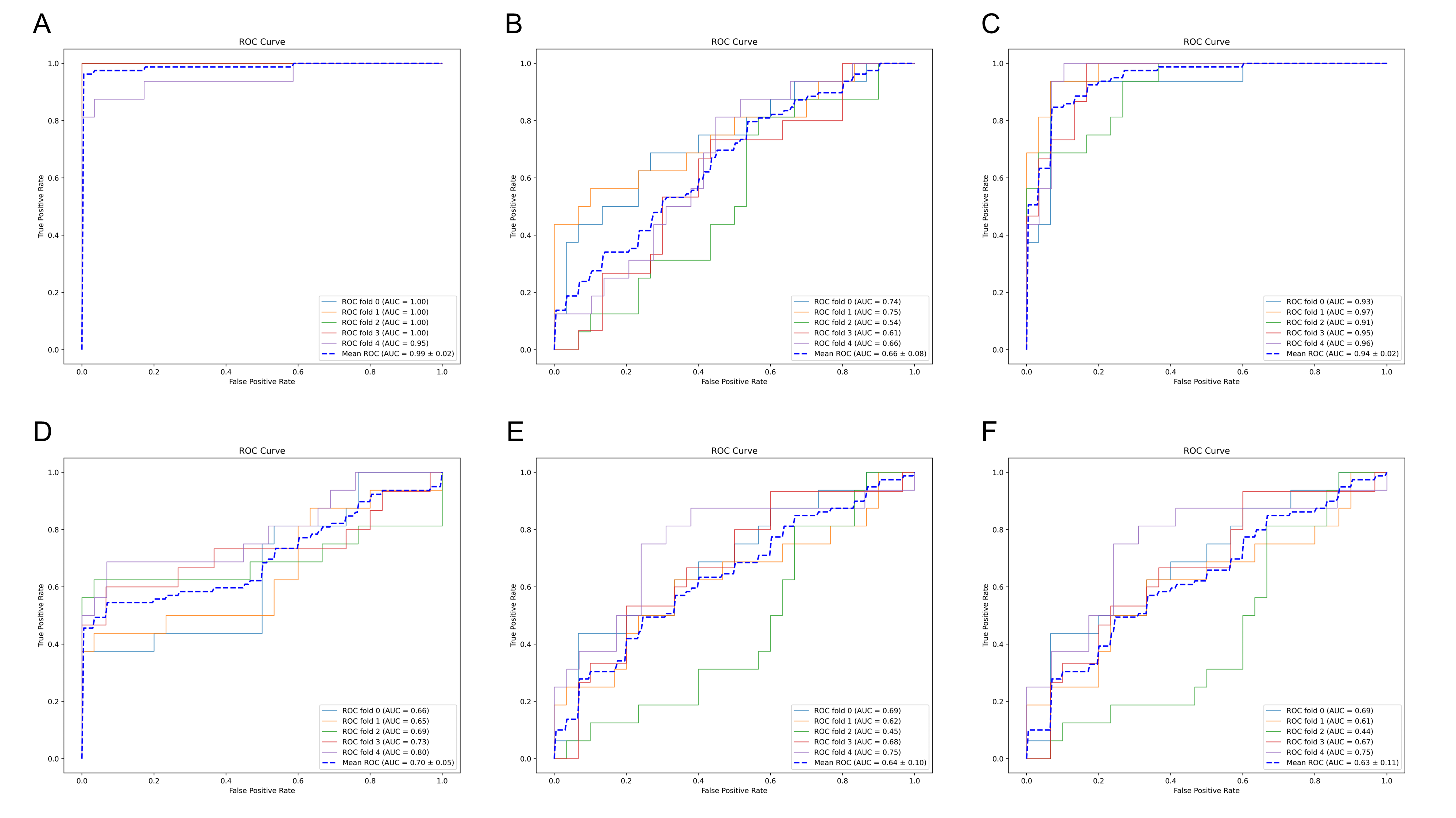


**Supplementary Figure S3.** Receiver operator characteristics (ROC) curves for five-fold cross-validation with different models in the training group.


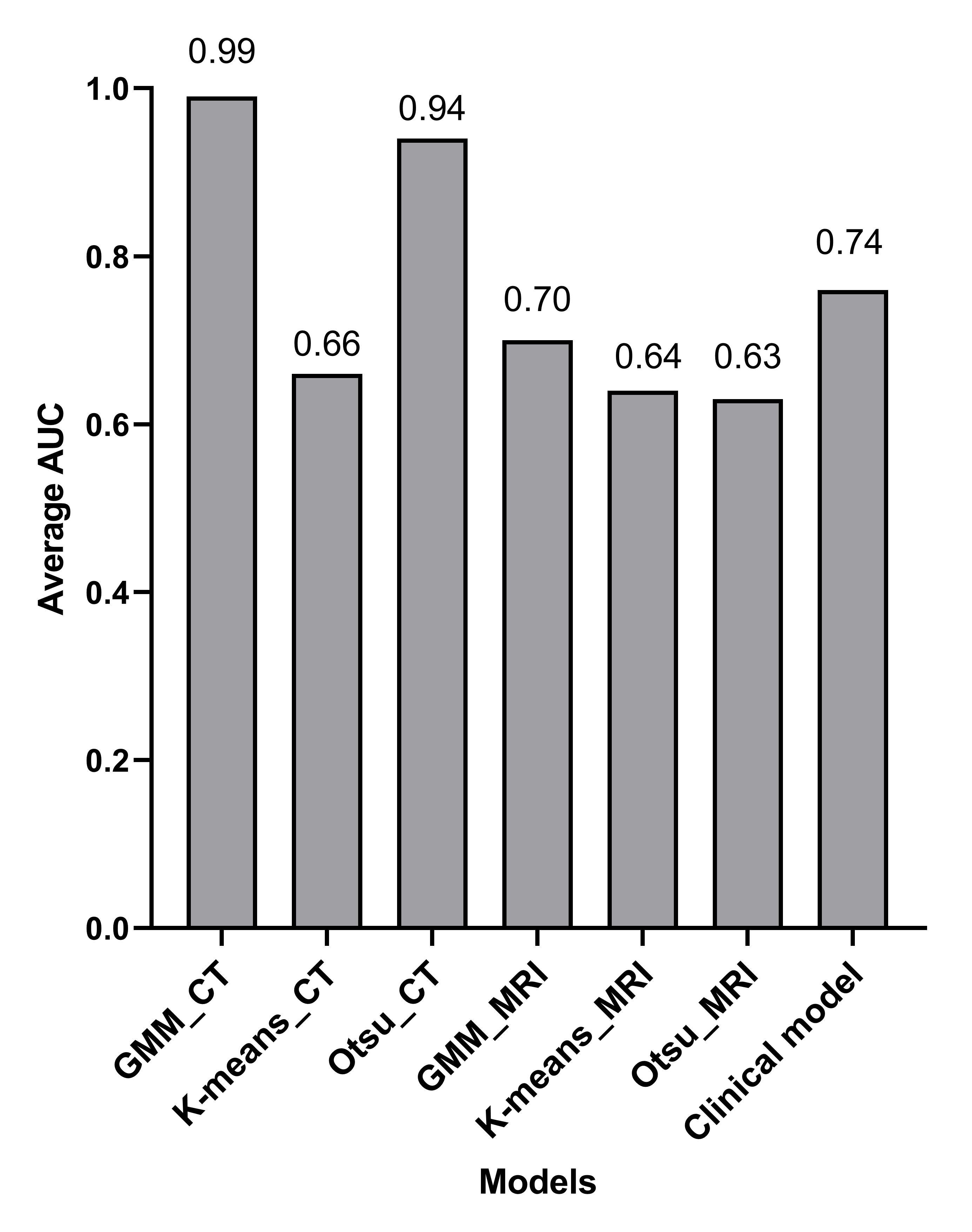


**Supplementary Figure S4.** Histograms of the average AUC values of the five-fold cross-validation of each model in the training group.
